# Perspectives of nursing professionals and older adults differ on aspects of care for older people after a nationwide improvement program

**DOI:** 10.1186/s12913-018-3114-x

**Published:** 2018-05-02

**Authors:** Lisanne Marlieke Verweij, Rik Wehrens, Lieke Oldenhof, Roland Bal, Anneke L. Francke

**Affiliations:** 1 Netherlands Institute of Health Services Research (NIVEL), Otterstraat 118-124, 3513 CR Utrecht, the Netherlands; 2 Erasmus School of Health Policy & Management, Erasmus University Rotterdam, Rotterdam, The Netherlands; 3 Amsterdam Public Health research institute, VU University Medical Center, Amsterdam, the Netherlands

**Keywords:** Perspectives, Older persons, Nursing professionals, National improvement program

## Abstract

**Background:**

The perspectives of nursing professionals might differ from those of older adults when it comes to care for older people. This cross-sectional study compares the views of older adults with the views of nursing professionals on the quality of care after a nationwide improvement program for care for older people was implemented (2008–2016) in the Netherlands.

**Methods:**

Questionnaire data were used from 385 nursing professionals (response rate 51%) that were part of the Nursing Staff Panel, a nationwide representative group of nursing staff, and working in home care, hospitals or general practices. Additionally, questionnaire data were used from 73 older adults (response rate 81%) who were involved in regional networks to discuss project proposals and to represent the voice of older adults in the nationwide improvement program. Participants were asked to evaluate care for older people with regard to collaboration between healthcare organizations and with regard to the tailored service, accessibility, and quality of care within their organizations and in the region in which they lived.

**Results:**

A majority of older adults (54%) and nursing professionals (61%) felt that collaboration with others had improved over the last few years. Approximately one third of the older adults stated that care for older people was tailored to fit individual needs and was accessible *most of the time* or *always*, as opposed to approximately two thirds of the professionals. Moreover, 17% older adults thought that the quality of care was *good*, compared with 54% of the nursing professionals. 77% of the nursing professionals and 94% of the older adults thought that improvements were still needed in care for older people, for example better integration of the different aspects of care and a more patient-centered approach.

**Conclusion:**

Older adults who were involved in networks of the improvement program generally gave a less positive evaluation of aspects of care for older people and its development than nursing professionals. Considering differences in the perspectives of key stakeholders is relevant for the development and evaluation of nationwide improvement programs, for a correct interpretation of findings, and for making appropriate recommendations.

**Electronic supplementary material:**

The online version of this article (10.1186/s12913-018-3114-x) contains supplementary material, which is available to authorized users.

## Background

As the population ages, the number of older adults suffering from multiple, complex health problems is increasing [1]. To keep care for older adults sustainable, contemporary healthcare policies are aimed at replacing residential long-term care by home-based care. This type of care requires an integrated and patient-centered approach [2]. At the same time, the World Health Organization observed that there is often little cooperation between healthcare organizations, and that available knowledge is often not used to maximum effect [3]. As a result, vulnerable older adults (adults with one or more chronic diseases) do not always receive the care they need.

To improve the quality of care for older people as well as reduce its costs, governments have established large-scale programs over the last 20 years [4]. In the Netherlands, for instance, the Ministry of Health, Welfare, and Sport commissioned a nationwide program between 2008 and 2016, called the National Care for Older people Program (NCOP, in Dutch: *Nationaal Programma Oudenzorg*) [5]. The main objective of the NCOP was to promote proactive, integrated healthcare for older adults with complex healthcare needs through regional networks of care providers, local improvement projects, the involvement of older adults in regional networks, and a national steering group [6]:

“***Regional networks***

*A main focus of the program was regional cooperation. The program funded the realisation of regional networks. All parties involved in (health)care for older adults were welcome to participate in these networks. For example, general practitioners, care and nursing homes, hospitals, home care services, health insurance companies, pharmacies and municipalities, but also older adults themselves. Networks could apply for a grant to fund projects aimed to improve the quality of care within their region.*

***Projects***

*A large part of the program budget was used to fund projects aimed to organise the care in innovative manners. Regional networks could submit proposals for projects as such. When doing so they were invited to think beyond the boundaries of existing legislation and types of funding. They could submit proposals for research on prevention possibilities, and/or improved diagnosis or treatment. The knowledge acquired was disseminated and implemented nationally. That phase of the program was also funded.*

***Involvement of older adults***

*The involvement of older adults was crucial for the success of the program. Their problems and wishes had to be the point of departure. Older adults were both regionally and nationally involved in discussions about new subjects and projects.”*

The program was coordinated by eight academic medical centers in the Netherlands and implemented in collaboration with local stakeholders in various healthcare sectors, such as general physicians and nursing professionals. The NCOP is different from many other major Dutch nationwide programs in that it was both a development program and a quality program [7–9]. That is to say, no specific interventions were chosen in advance for implementation; the regional networks could choose themselves which innovations or areas of research they wanted to develop or implement. Their project proposals were evaluated in a succession of subsidy rounds. Often the innovations and research projects had to do with transmural care, case management or innovations to connect healthcare and welfare services more closely. The NCOP’s aims were increased self-reliance and independence among older adults, a greater retention of function in older adults, less reliance on care services, and a reduced risk of initiating care and treatments that are unnecessarily burdensome [10]. In addition to these clinical outcomes for older adults, the NCOP was expected to result in more attention in health services for care of older adults in general, in more and better regional collaborations, and in care that is better tailored to individual needs, more accessible, and of higher quality. Details of the effectiveness of the individual interventions have been reported in other publications [11–13].

How the NCOP and care for older adults in general are appreciated might differ between those providing care and those receiving that care. In any field, improving performance depends on having a shared goal that unites the interests and activities of all stakeholders. In health care, however, stakeholders often have conflicting goals, including access to services, high quality, cost containment, safety, convenience, patient-centeredness, and satisfaction. Lack of clarity about goals has led to divergent approaches, and may slow progress in performance improvement [14]. Various studies show that care professionals can indeed differ in their views from care recipients. For example, studies have shown that professionals evaluate the general wellbeing and health outcomes of older adults more positively than older adults themselves [15]. Other studies have shown that residents’ scores are significantly higher than ratings by staff in evaluations of dementia residents’ quality of life [16]. Moreover, older persons recognize only a few conditions as problems themselves, compared to professionals’ comprehensive assessment of geriatric conditions [17]. Previous evaluations of large national programs paid little attention to the fact that caregivers and care recipients may hold different views about whether the quality of care has improved [7–9]. Given the possible differences in perspectives between professional caregivers and care recipients when it comes to health and healthcare-related issues, it may be important to take both perspectives into account when evaluating the success of a care improvement program. This paper presents the evaluation of the National Care for the Older adults Program from both perspectives and addresses the following research questions:

- How do nursing professionals and older adults evaluate aspects of care of older adults after a nationwide improvement program?
- Do evaluations by older adults and nursing professionals differ?

## Methods

### Participants

The participants were nursing professionals and older adults. Nursing professionals are currently the largest group of care professionals in the Netherlands for older adults and form the main contact point for the vulnerable older adults in daily life. Primary care nurses in particular are key figures in local home-based care, and form the linking pins between professionals in social and medical disciplines [16].

Nursing professionals were recruited through the Nursing Staff Panel, a nationwide representative group of nursing staff [18]. All the members of this panel deliver direct patient care and voluntarily fill out periodic questionnaires on current healthcare topics. Panel members were eligible to participate in this specific survey if they met the following inclusion criteria:

1. caring for vulnerable older adults, i.e. older adults with one or more chronic conditions or dementia;
2. working in a home-care service, general or university hospital or in a general practice;
3. being a Registered Nurse (educated to bachelor or vocational level), certified nurse assistant (vocational level) or working as a practice nurse in a general practice.

Of the 741 nursing professionals in the Nursing Staff Panel who were eligible to participate, 385 (52%) completed our online questionnaire.

Older adults who were involved in regional networks initiated in the improvement program were recruited through the coordinators of the eight academic medical centers that participated in the NCOP. From 2008 on, a total of 125 older adults were selected and trained to actively participate in regional collaborative networks to discuss new projects and represent the voice of older adults. The selection criteria were the ability to read and understand project documentation, and the capacity to travel to meeting locations. For this cross-sectional study, the coordinators sent detailed information on the study and the link to the online questionnaire per email. The coordinators were able to reach 91 older adults who were able to participate in this study, of whom 73 (81%) actually participated.

### Questionnaires

The questionnaires were based on the periodic questionnaires sent to the Panel and adapted with specific questions for this study. The questionnaires were pretested among 11 nursing professionals and four older adults for comprehensibility, completeness, time to complete, and any further comments regarding the questionnaire. The questionnaires were finalized based on their comments. The key questions are provided in Additional file 1. The questionnaires were sent to the participants digitally for efficiency reasons. A paper-based questionnaire was sent to one older adult without Internet, after which the completed questionnaire was uploaded by the research team. Data collection took place between April and August 2016. Reminders were sent after 2 weeks and after 4 weeks.

The questionnaire that nursing professionals and older adults were asked to complete had three domains: 1) background characteristics; 2) aspects of collaboration; 3) tailored care, accessibility, and quality of care for older adults. Participants were generally asked to choose from the answer categories ‘yes’, ‘no’ and ‘do not know’ unless specified otherwise, and they could provide additional remarks. Details on the domains are provided below.

#### Background characteristics

Both professionals and older adults were asked to state their age, sex, and the region in which they lived. To gain insight in the societal involvement of the NCOP older adults, we asked the older adults how many associations for older adults they were involved in and for how long. In addition, nursing professionals were asked about their workplace (home care service, general or university hospital, or general practice), position (clinical and/or management) and years of working experience. The professionals were additionally asked if they felt equipped to provide tailored care to older adults with complex care needs. Furthermore, the professionals were asked whether they had undertaken any training activities in recent years with regard to care for older adults with complex health needs and if these training activities had met their needs. Finally, the professionals were asked if they were familiar with the National Care for the Older adults Program.

#### Collaboration

The professionals were asked to state if they collaborated in a regional network and/or with other professionals in the region. All participants were additionally asked if they felt that collaboration had improved, and whether care for older adults had received more attention within their workplace (professionals) or within their region (older adults) over the last few years. Finally, we asked participants about the perceived need for changes to improve care for older adults.

#### Tailored care, accessibility, and quality of care

Participants were asked whether they thought that the care and support for older adults with complex healthcare needs was tailored to their specific needs and whether it was accessible. *Tailored care* was defined as care or support that matches the needs, wishes and abilities of older adults. *Accessibility* was defined as timely and accessible care or support without great barriers, when older adults are in need of care or support. Older adults with *complex care needs* were defined as older adults with one or more chronic conditions or dementia. Finally, participants were asked to provide a general rating for the quality of care and support that older adults with complex healthcare needs receive. *Quality of care* was undefined to allow for a personal view of participants.

### Analyses

Descriptive statistics were performed to explore the participants’ answers. Chi-square tests and t-tests were performed to detect possible differences between evaluations by older adults from different regions in the Netherlands, and between nursing professionals who were familiar with the NCOP and those who were not. Analyses were performed using STATA 13.

## Results

### Background characteristics

The participants’ background characteristics are presented in Table 1. On average, older adults involved in the nationwide improvement program were 74 years of age (SD 6; range 58–91) and the majority were female (60%, *n* = 44). One region was overrepresented among the older adults (*n* = 24), but there was an even distribution across the other seven regions. Most older adults (71%) were members of two or more associations for older adults, for 12 years on average.

**Table 1:** Descriptive statistics of older adults and nursing professionals

Older adults (*n* = 73)		
Sex		
Male	29	(40%)
Female	44	(60%)
Age	74	(mean; SD 6; range 58–91)
Region		
1	7	(11%)
2&8	9	(14%)
3	6	(9%)
4	7	(10%)
5	24	(36%)
6	5	(8%)
7	8	(12%)
Member of association for older adults, years on average		
two	52	(71%), 12
three	30	(41%), 7
four or more	16	(22%), 5
Nursing professionals (*n* = 385)		
Sex		
Male	22	(6%)
Female	362	(94%)
Age	49	(mean; SD 10; range 21–64)
Workplace		
General hospital	83	(22%)
University hospital	8	(2%)
care services	240	(62%)
General practice	53	(14%)
Position		
Clinical	330	(85%)
Clinical and/or management	54	(14%)
Working experience (years)	24	(mean; SD 11; range 1–47)
Feeling equipped to provide tailored care to older adults with complex care needs		
Totally disagree	1	(0.3%)
disagree	24	(7%)
neutral	66	(18%)
agree	218	(60%)
Totally agree	53	(15%)
Undertook training activities last years concerning older persons with complex care needs		
No	113	(31%)
yes	249	(69%)
Did training activities meet your needs?		
Do not know	133	(37%)
No, because	85	(23%)
yes	144	(40%)
Familiarity with NCOP		
no	290	(80%)
yes	72	(20%)

Nursing professionals were 49 years old on average (SD 10; range 21–64) and usually female (94%). The majority of nursing professionals (62%) were based in home-care services, followed by 22% based in general hospitals, 2% in academic hospitals, and 14% working as practice nurses in general practices. On average, nursing professionals had 24 years of experience as a care provider (SD 11; range 1–47). 75% of the nursing professionals felt able to deliver tailored care to older adults and informal caregivers. Most professionals (69%) had received training in recent years on topics related to older adults with complex care needs; the nature of the training varied from reviewing case histories and conferences to specific topics. 23% felt that training possibilities in the region were insufficient, compared with 40% who felt there were sufficient possibilities and 37% who answered ‘do not know’. 20% of the professionals were familiar with the NCOP, mainly via trade magazines, conferences, and training, sometimes via the media and the Internet, and in a few cases because of direct involvement in the program.

### Collaboration

**Fig. 1:**
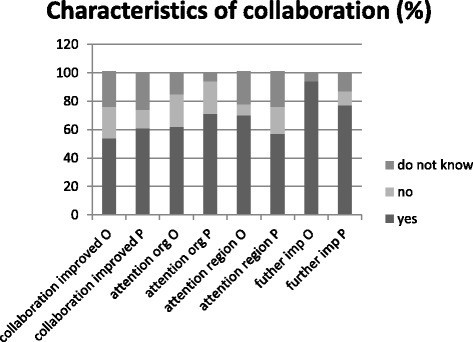
Characteristics of collaboration as perceived by nursing professionals (P) and older adults (O)

The perceived characteristics of collaboration are shown in Fig. 1. A slight majority of older adults (54%) and nursing professionals (61%) thought that collaboration with other professionals had improved over the last few years. Of the older adults, 62% stated that more attention had been paid to older adults with complex care needs over the last few years within their organization, compared to 71% of the nursing professionals. Moreover, 70% of the older adults felt older adults with complex care needs were receiving more attention within their *region*, compared to 57% of nursing professionals. 94% of the older adults and 77% of the nursing professionals felt that further improvements still needed to be made in care for older adults, such as better coordination of the different aspects of care (e.g. health care, welfare, housing, and participation), and a more patient-centered approach (Table 2).

**Table 2:** Areas of care that need improvement, typically explained by nursing professionals and older adults

Nursing professionals stated: “More collaboration than is now the case, increase knowledge (especially on recognizing vulnerability) and more action towards preventing vulnerability.”	
“Less segregation of organisations and finances. It is difficult to keep track of changing names of organisations and staff. Appointing one case manager that provides overview and arranges appropriate care would be helpful.”	
“Talking to older adults at their kitchen table and asking what they need and how they approach life.”	
Older adults stated: “Better inform older adults, encourage prevention, more low-threshold walk-in services, empower patiënts, allow fulfilled-life discussions, battle loneliness and malnutrition, etc.”	
“Let questions of (older) citizens be leading in care from sincere interest, allowing for a reduction of over- and under treatment. Thus tailored care.”	

### Tailored care, accessibility, and quality of care.

**Fig. 2:**
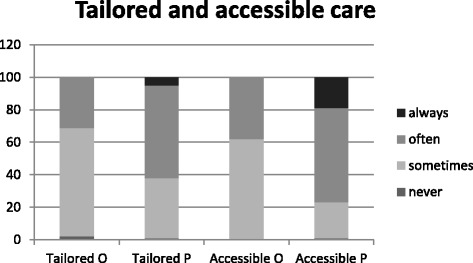
Tailored and accessibility of care as perceived by nursing professionals (P) and older adults (O)

**Fig. 3:**
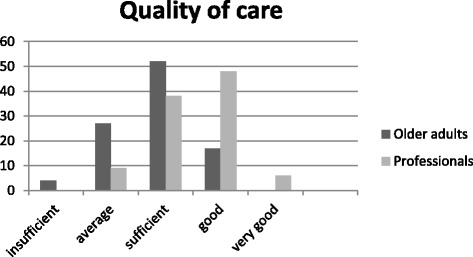
Quality of care as perceived by nursing professionals and older adults

Participants’ perceptions of tailored care, accessibility, and quality of care are presented in Fig. 2 and Fig. 3. Of the older adults, 31% stated that care for older adults was tailored to fit needs *most of the time* or *always*, compared with 62% of the professionals. Furthermore, 38% of the older adults stated that care was accessible *most of the time* or *always,* whereas 77% of the professionals gave these answers. Moreover, 17% of older adults felt that the quality of care was *good*, as opposed to 54% of the nursing care professionals.

### Comparison of results

No differences were found in answers between older adults who resided in different regions in the Netherlands. Nursing professionals who were familiar with the NCOP (20%) consistently gave more positive answers to all questions compared to those who were not familiar with the NCOP (data not shown).

## Discussion

This study describes the evaluations by nursing professionals and older adults regarding care for older adults after a nationwide program was implemented in the period 2008–2016. Overall, over half of the nursing professionals and older adults perceived an increase in collaboration and in the attention being paid to care for older adults over the past few years. Nevertheless, most nursing professionals and almost all older adults stated that further improvements should be made to care for older adults in the Netherlands. According to the participants, there was room for improvement in particular through better coordination of the different aspects of care for older adults and by developing a more patient-centered approach. A clear contrast was seen between the perceptions of older adults and those of nursing professionals in their evaluations of tailored care, accessibility, and the overall quality of care. Older adults gave a less positive evaluation of these aspects of care than nursing professionals did. Finally, an important finding is that nursing professionals who were familiar with the NCOP (20%) consistently gave more positive answers to all questions compared to those who were not familiar with the NCOP.

In general, the results that were obtained in our evaluation study are in line with the results of earlier evaluation studies of national improvement programs in care for older adults. The national Dementia Program developed a national format for integrated dementia care through multidisciplinary network structures. The national Dementia Program evaluation concluded that more collaboration and more satisfaction had been achieved, according to participants [7]. Another national program, ‘Visible Link’, which aimed to increase collaboration among primary caregivers, healthcare providers, and patients, reported more collaboration and higher satisfaction with care as well [8]. However, these program evaluations did not evaluate possible differences between key stakeholders, that is caregivers and care recipients [7–9]. This study has clearly shown that the perspectives of professionals providing care differ from those of the recipients of care, especially if the professionals are familiar with the care improvement program. The older adults might have been more positive about the increased attention for older adults with complex care needs within their *region* because of their participation in these regional networks. Moreover, the nursing professionals who were familiar with the program may have been consistently more positive about developments in care for older adults, because they were more motivated and interested in healthcare improvements [19]. On the other hand, older adults were more negative in their perception of tailored care, accessibility, and the quality of care. This suggests an important distinction between the relatively *active* older adults who participated in the national program, and older adults who are dependent on care and support. Vulnerable older adults in institutionalized care, for example, are known to be satisfied and report few complaints because of their dependency on health care [19, 20]. Algilani et al. further argue that older adults as a group are heterogeneous in terms of their preferences and views on health and should thus be approached as such in the healthcare setting [21]. Moreover, comparison of health indicators among different groups of stakeholders (amongst which healthcare providers, patients, policy makers, and researchers) showed that patients considered all dimensions important, including spirituality and social dimensions of health, thus preferring a broad concept of health, whereas others assessed health more narrowly and as primarily bio-medical [22].

Relying on a one-sided evaluation of care improvements may impede a correct interpretation of findings. This may jeopardize appropriate recommendations with regard to healthcare policies [17]. Moreover, discrepancies between patients’ perceptions of health and the usually narrower perception of health by other stakeholders require attention in view of the prevailing policy trend towards ‘patient-centered care’. Especially when ‘shared decision-making’ is practiced, accounting for different views may prevent misunderstandings, and improve communication in medical practice, and could even imply that areas other than the medical domain and different groups of professionals might be relevant [22].

The strengths of this study are the large national sample of key stakeholders and the comparison of possible differences in perspectives. The differences were not tested statistically due to slight differences in the focus of the questions. Moreover, as the NCOP was one development amongst many others since 2008 in care for older adults, including national programs, changes in health care, regional variances and legislation, causality for any perceived improvements cannot be assigned. A consideration is that the older adults who participated in the national improvement program, and thus in this study, were relatively active older adults and not necessarily vulnerable older adults with complex care needs. It is a common phenomenon in large-scale programs to select participants who are best able to participate [23]. As a consequence there may have been an underrepresentation of vulnerable adults. Moreover, due to the differences in sample size, external validity may be limited. It is possible that older adults in general (e.g. a random sample) have different opinion, strengthening our recommendation to include perspectives of different stakeholders. Finally, pre-measurement data were not available, which may have led to recollection bias. To overcome this, we structured the questionnaire to focus first on aspects of care for older adults, followed by specific questions about the national program.

## Conclusion

This study has shown that the different parties involved – professionals giving care and older adults receiving care – had different perspectives on the quality of care provided and the improvements achieved in a national development program in care for older adults. In general, older adults were less positive about the improvements in care achieved by the program than care professionals. This indicates that when evaluating improvement programs it is not sufficient to focus exclusively on the opinions of care professionals and other professional stakeholders; it is necessary to also include the voice of the target audience, i.e. those who are receiving the care.

## Additional file

Additional file 1: Key questions asked to nursing professionals and older adults. (DOCX 36 kb)

## Abbreviation

NCOP: National Care for the Older adults Program
