# Supplementary material for: Perspectives of nursing professionals and older adults differ on aspects of care for older people after a nationwide improvement program
Source: BMC Health Serv Res. 2018 May 2;18:321. doi: 10.1186/s12913-018-3114-x (PMC5932835; doi:10.1186/s12913-018-3114-x)
Supplement: Supplementary file 1 — Key questions asked to nursing professionals and older adults. (DOCX 36 kb) [file 12913_2018_3114_MOESM1_ESM.docx]

**Additional files**

Additional file 1. Key questions asked to nursing professionals and older adults.

*Domain: Collaboration*

Older adults with complex care needs are defined as older adults with one or more chronic conditions or dementia.

1. Do you collaborate in a regional network and/or with other professionals in the region?
   - No
   - Yes
2. Do you feel that collaboration with other professionals other than your direct colleagues (professionals) / organisations (older adults) in your region has improved over the last few years in the care and support to vulnerable older adults?
   - I do not know
   - No
   - Yes
3. Do you feel that care and support to vulnerable older adults has received more attention in your organisation (professionals) / or in your region (professionals and older adults) over the last few years?
   - I do not know
   - No, that has not changed
   - No, care and support to vulnerable older adults has received *less* attention within my organisation
   - Yes, care and support to vulnerable older adults has received *more* attention within my organisation
4. Do you feel a need for change in order to improve care and support to vulnerable older adults in your organisation (professionals) / or in your region (professionals and older adults)
   - I do not know
   - No
   - Yes
5. Changes that should be made to improve care and support to vulnerable older adults in my organisation (professionals) / or in my region (professionals and older adults) are: ….

*Domain: Tailored care*Tailored care is defined as care or support that matches the needs, wishes and abilities of older adults.

1. In your experience, is care and support in your organization (professionals) / in your region (older adults) tailored to vulnerable older adults?
   - never
   - sometimes
   - often
   - always

*Domain: Accessibility of care*Accessibility is defined as timely and accessible care or support without great barriers, when older adults are in need of care or support.

1. In your experience, is care and support in your organization (professionals) / in your region (older adults) accessible to vulnerable older adults?
   - never
   - sometimes
   - often
   - always

*Domain: Quality of care*

1. How would you rate the quality of care and support that vulnerable older adults generally receive in your organization (professionals) / in your region (older adults)?
   - very good
   - good
   - sufficiënt
   - average
   - insufficiënt
